# Evolution of NETosis markers and DAMPs have prognostic value in critically ill COVID-19 patients

**DOI:** 10.1038/s41598-021-95209-x

**Published:** 2021-08-03

**Authors:** Joram Huckriede, Sara Bülow Anderberg, Albert Morales, Femke de Vries, Michael Hultström, Anders Bergqvist, José T. Ortiz-Pérez, Jan Willem Sels, Kanin Wichapong, Miklos Lipcsey, Marcel van de Poll, Anders Larsson, Tomas Luther, Chris Reutelingsperger, Pablo Garcia de Frutos, Robert Frithiof, Gerry A. F. Nicolaes

**Affiliations:** 1 Department of Biochemistry, Cardiovascular Research Institute Maastricht (CARIM), Maastricht University, P.O. Box 616, 6200 MD Maastricht, the Netherlands; 2 Department of Surgical Sciences, Section for Anaesthesia & Intensive Care, Uppsala University, Uppsala, Sweden; 3 Department of Cell Death and Proliferation, IIBB-CSIC, IDIBAPS, and BCLC, CIBEREHD, Barcelona, Spain; 4 Department of Medical Cell Biology, Integrative Physiology, Uppsala University, Uppsala, Sweden; 5 Department of Medical Sciences, Clinical Microbiology, Uppsala University, Uppsala, Sweden; 6 Cardiology Department, Hospital Clinic Barcelona and CIBERCV, Barcelona, Spain; 7 Department of Intensive Care Medicine, Maastricht University Medical Centre MUMC+), Maastricht, the Netherlands; 8 Department of Cardiology, Maastricht University Medical Centre, MUMC+), Maastricht, the Netherlands; 9 Hedenstierna Laboratory, Anaesthesiology and Intensive Care Medicine, Department of Surgical Sciences, Uppsala University, Uppsala, Sweden; 10 Department of Surgery, Maastricht University Medical Centre (MUMC+), School for Nutrition and Translational Research in Metabolism (NUTRIM), Maastricht University, Maastricht, the Netherlands; 11 Department of Medical Sciences, Clinical Chemistry, Uppsala University, Uppsala, Sweden; 12 Department of Cell Death and Proliferation, IIBB-CSIC, IDIBAPS and CIBERCV, Barcelona, Spain

**Keywords:** Respiratory tract diseases, Disease-free survival, Acute inflammation, Sepsis, Neutrophils, Infection, Viral infection

## Abstract

Coronavirus disease 19 (COVID-19) presents with disease severities of varying degree. In its most severe form, infection may lead to respiratory failure and multi-organ dysfunction. Here we study the levels and evolution of the damage associated molecular patterns (DAMPS) cell free DNA (cfDNA), extracellular histone H3 (H3) and neutrophil elastase (NE), and the immune modulators GAS6 and AXL in relation to clinical parameters, ICU scoring systems and mortality in patients (n = 100) with severe COVID-19. cfDNA, H3, NE, GAS6 and AXL were increased in COVID-19 patients compared to controls. These measures associated with occurrence of clinical events and intensive care unit acquired weakness (ICUAW). cfDNA and GAS6 decreased in time in patients surviving to 30 days post ICU admission. A decrease of 27.2 ng/mL cfDNA during ICU stay associated with patient survival, whereas levels of GAS6 decreasing more than 4.0 ng/mL associated with survival. The presence of H3 in plasma was a common feature of COVID-19 patients, detected in 38% of the patients at ICU admission. NETosis markers cfDNA, H3 and NE correlated well with parameters of tissue damage and neutrophil counts. Furthermore, cfDNA correlated with lowest p/f ratio and a lowering in cfDNA was observed in patients with ventilator-free days.

## Introduction

In severe cases, COVID-19 disease develops into acute respiratory distress syndrome (ARDS), an acute lung injury causing patients to be dependent of ventilator support, which may be accompanied by development of multiple organ failure (MOF)^1^. Mortality is seen primarily in patients over the age of 65^2–5^ and is highest for infected individuals with underlying comorbidities such as hypertension, cardiovascular disease or diabetes^6–8^. For patients who are taken into the intensive care unit (ICU), a high SOFA (sequential organ failure assessment) score and increased levels of fibrin D-dimers have been reported^9^ to associate with poor prognosis. Thromboembolic complications develop in 35–45% of COVID-19 patients^10^, including thrombotic microangiopathies and disseminated intravascular coagulation (DIC) reminiscent of bacterial sepsis. Yet, COVID-19 has distinct features^11^ that point at a somewhat different pathological mechanism. The involvement of immune regulatory and hemostatic pathways appears evident, and recent findings have confirmed that the innate immune system and more in particular neutrophil extracellular traps (NETs) play a role in COVID-19 disease pathogenesis. NETs, networks of DNA fibers that are decorated with proteins such as histones and elastase, are released from neutrophils to bind and neutralize viral proteins, bacteria and fungi^12^. While extracellular histones and NE serve a protective, antimicrobial function, they are potentially harmful to the host.

NETs are abundant in lung capillaries^13^ and are known to be pro-coagulant due to their intrinsic capacity to activate platelets^14^.

Excessive NET production, initiated by several pathways that also include complement activation^13^, results in collateral damage to lung tissues, a disturbed microcirculation of the lung^15^, loss of alveolar-capillary barrier function and further release of pro-inflammatory cytokines^16^.

During the preparation of this work it was reported that cellular components that are released upon cellular disruption, so-called damage associated molecular patterns (DAMPs) and NETosis are involved in COVID-19 disease^17^^18^. This is fully in line with the observation that in ARDS, NETs contribute to disease progress^19^. Extracellular histones are cytotoxic DAMPs irrespective of their origin. They may appear during NETosis^12,14,20^ or originate from damaged tissues^21^, while cell free DNA (cfDNA) and the protease neutrophil elastase (NE) are released concomitantly^22^. Cellular free deoxyribonucleic acid (cfDNA) and histones promote proinflammatory cytokine release^23,24^. Histones have been shown to activate and recruit leukocytes^25^, damage alveolar macrophages^26^, activate erythrocytes^27^, epithelial and endothelial cells, in particular pulmonary endothelial cells^28–30^. If not cleared from circulation, cfDNA as well as histones facilitate severe systemic inflammation and worsen the clinical condition^31,32^. Presence of NE in plasma is associated with exacerbations, lung function decline and disease severity in patients with chronic obstructive pulmonary disease (COPD), bronchiectasis and cystic fibrosis^33–35^ and decrease of NE levels in bronchiectasis patients improved lung function and airway inflammation^36^.

At the same time that it provides a first line of defense against infections, the innate immune system initiates self-control responses to prevent damage to the host. One mechanism involved in early immunomodulation is the growth arrest-specific 6 (GAS6)/TAM ligand/receptor system^37,38^. The GAS6/AXL axis regulates the immune response by modulating cytokine production, inducing a reparative cellular response and by mediating efferocytosis, removing irreversibly damaged cells. The system also provides a mechanism of regulating endothelial and platelet activation and interaction^39^. Plasma concentrations of GAS6 and AXL increase in a diverse spectrum of inflammatory conditions^40^, including sepsis and septic shock; but also systemic inflammatory response syndrome (SIRS) without infection^41^. In several studies, GAS6 at IC admission correlated with severity of organ damage (i.e. SOFA) or with damage of specific organs^41–45^. This is also the case in viral infections^46^. These studies illustrate the modulatory role of the innate response provided by GAS6 and suggest that the presence of these components in plasma could be an early event in the orchestration of the immune response to viral infections.

cfDNA, extracellular histones and GAS6 are implicated in regulation of inflammatory and hemostatic pathways in the context of severe viral infections and ARDS, all of which are implicated in COVID-19. While other studies have reported the presence of DAMPs and NETosis markers in smaller COVID-19 populations, here we study a group of 100 severely ill COVID-19 patients admitted to the intensive care unit (ICU). Our hypothesis was twofold:

First, cfDNA, NE, histones and GAS6/AXL are activated in severe COVID-19. Second, cfDNA, NE, histones and GAS6/AXL are related to the severity of illness and reflect organ dysfunction in severe COVID-19.

## Materials and methods

### Subjects and sampling

All adult patients with confirmed or suspected COVID-19 admitted to the ICU between March 21, and June 6, 2020 were screened for eligibility. The COVID-19 diagnosis was established by PCR detection of severe acute respiratory syndrome coronavirus 2 (SARS-CoV-2) E and N-genes in nasopharyngeal swabs according to previously described protocols^47^. For all ICU patients severity of illness and organ dysfunction were recorded by monitoring basal clinical functions and calculation of the simplified acute physiology score-3 (SAPS-3), sequential organ failure assessment score (SOFA), and intensive care unit acquired weakness (ICUAW) scores. Plasma samples were collected from 100 consecutive patients. We further used 11 previously included ICU-patients without COVID-19 as ICU-controls and included 15 healthy control subjects who were healthy university employed volunteers.

For a subset of patients, longitudinal plasma samples were available (n = 33), taken between days 1 and 12, which allowed analysis of time-dependent development of plasma values for the markers measured. For other patients, longitudinal sampling could not be performed due to mortality and/or limitations in logistic capacity at the peak of ICU occupancy.

The first blood sample (in citrate buffer) collected after a patient was admitted to the ICU was used as baseline measurement. Platelet poor plasma (PPP) was prepared by centrifugation of the blood for 10 min at 3000×*g* at 4 °C, after which the supernatant was carefully pipetted, whilst taking care not to disturb the cell-containing lower layer by keeping a generous margin from the buffy coat. Platelet poor plasma (PPP) was aliquoted and snap-frozen until use. The healthy control PPP was prepared by the same method as the patient plasma.

### Quantitation of cell-free DNA

Cell-free DNA (cfDNA) was quantitated from plasma essentially as described earlier^48^ using a real-time PCR-based assay. In short, plasma samples were diluted eightfold in water to result in a final assay dilution of 40 times. Reactions were performed in 96-well plates (Roche) employing a LightCycler 480 qPCR machine (Roche). Reaction volumes contained 5 μL of TATAA Probe GrandMaster Mix/no ROX (TATAA Biocenter), 0.5 μL TATAA Alu-60 assay probes (TATAA Biocenter), 2.5 μL H_2_O and 2 μL of sample. Amplification consisted of a pre-denaturation step 2 min at 95 °C, to activate the DNA Polymerase in the master mix. Followed by 40 cycles of denaturation at 95 °C for 5 s, annealing at 60 °C for 10 s and extension at 60 °C for 30 s. A calibration range (from 1 to 300 ng/μL) using purified and quantitated DNA standard prepared as described^49^ was included in each analysis, facilitating the direct correlation of Ct values to DNA concentration.

### Quantitation of NE, sAXL, and GAS6 in Plasma

NE, soluble AXL (sAXL), and GAS6 levels in plasma were determined by the ELISA technique using commercial kits from R&D systems (DuoSet ELISA, Bio-techne, Minneapolis, USA) according to the manufacturer’s instructions. For determination of sAXL, the plasma form of the AXL cellular receptor, and GAS6, plasma samples were diluted 1:40 and 1:200 for NE. All samples were determined in duplicates*.*

### Analysis of extracellular histone H3

Extracellular histone H3 levels were determined using a semi-quantitative method previously described^50,51^. Briefly, plasma dilutions were subjected to SDS-PAGE gel electrophoresis and transferred to PVDF membranes (Bio-Rad Laboratories, Hemel Hempstead, UK) using semi-dry blotting. Membranes were blocked and incubated with primary anti-H3 antibody, o/n at 4 °C, (sc-8654-R, Santa Cruz Biotechnology, Heidelberg, Germany), followed by a secondary biotin-conjugated IgG for 30 min at RT (ab97083, Abcam, Cambridge, UK) and a streptavidin–biotin/alkaline phosphatase complex (Vectastain ABC-Alkaline Phosphatase for 30 min at RT, Vector Laboratories, Burlingame, USA). Histone H3 bands were detected by luminescent ECL substrate (Advansta, San Jose, USA). Resulting band densities were quantified by ImageQuant TL software (GE Healtcare, Little Chalfont, UK), as compared to known concentrations of purified calf thymus H3 (Roche, Basel, Switzerland). This analysis is independent of frequently observed cross reactivity of histone antibodies with non-histone plasma proteins and allows the inspection of potential in vivo histone proteolytic processing.

### Statistical analysis

Graphpad Prism version 8 (Graphpad Software Inc., La Jolla, CA, USA) and SPSS Statistics version 26 (SPSS Inc., Chicago, IL, USA) were used for statistical analysis. We employed a Shapiro–Wilk test to inspect normality of data. Parametric data are presented as mean (SD) or geometric mean (95% CI) for log-transformed data unless stated. Paired and unpaired t-test and one-way analysis of variance (ANOVA) were used to compare variables. Non-parametric data are presented as median and inter quartile range (IQR). The Kruskal Wallis test was used to analyze variance, Mann–Whitney U tests to test unpaired groups, and Wilcoxon matched-pairs signed rank to test paired groups. Spearman’s rank-order test was used to calculate correlations. Ventilator free days was calculated based on^52^; number of days from day 1 to day 28 on which a patient breathed without assistance, if a patient dies or required more than 28 days of mechanical ventilation the value is 0. With this method also the vasoactive and dialysis free days were calculated. Receiver operating characteristic (ROC) curves were used to determine the accuracy of plasma markers to predict 30-day mortality. An optimal cutoff was determined when the Youden index reached the maximum value. A Kaplan–Meier plot and log-rank test (Mantel-Cox) of 30-day mortality was constructed based on the cutoff value of a plasma marker. P-values were considered significant if p < 0.05 unless stated; Significance is indicated as *p < 0.05, **p < 0.01, ***p < 0.001.

### Ethics approval and consent to participate

The study was approved by the Swedish National Ethical Review Agency (EPM; No. 2020-01623). Informed consent was obtained from the patient, or next of kin if the patient was unable give consent. The Declaration of Helsinki and its subsequent revisions were followed. The protocol of the study was registered (ClinicalTrials ID: NCT04316884).

## Results

Of all ICU patients included, 100 patients had a confirmed COVID-19 diagnosis and 11 patients did not have COVID-19 and were used as ICU control patients. We obtained a total of 470 plasma-samples from the 100 patients with confirmed COVID-19, while 18 samples from 11 patients were obtained from the ICU control group.

The baseline (day 1) characteristics of the 100 ICU patients are shown in Table 1. Between COVID-19 and non-COVID-19 patients, there were no significant differences in age, gender or body mass index (BMI) between both ICU groups. To allow comparison with a healthy population, a healthy control group was further included in our analyses. The healthy control group consisted of 15 individuals with a median age of 32 (IQR 24–37) of which 7 were male (47%).

**Table 1:** Demographic and baseline characteristics of 111 patients on admission to the Intensive Care Unit.

	ICU Covid-19 n = 100	ICU Control n = 11	*P*
Age, yrs, median	62 (51–73)	70 (59–75)	0.225
Gender, male N	74 (74)	5 (45.5)	0.075
BMI	29 (26–33)	27.7 (25.4–31.8)	0.675
Respiratory rate, breaths/min	28 (23–36)	15 (15–17)	< 0.001
Heart rate, beats/min	89 (77–100)	80 (72–92)	0.179
MAP, mmHg	89 (79–97)	80 (67–86)	0.012
Temperature, °C	38.0 (37.5–38.7)	36.4 (36.4–36.7)	< 0.001
Diabetes, yes	29 (29)	2 (18.2)	0.725
Hypertension, yes	52 (52)	4 (36.4)	0.325
Heart failure, yes	5 (5)	1 (9.1)	0.474
Ischemic heart failure, yes	12 (12)	0 (0)	0.605
Vessel disease, yes	17 (17)	1 (9.1)	0.689
Malign disease, yes	6 (6)	11 (100)	< 0.001
HIPEC surgery, yes	0 (0)	11 (100)	< 0.001
PaO2/FiO2-ratio, mmHg	138.1 (116.3–178.1)	271.5 (227.3–373.9)	0.001
Ventilation, yes	100 (100)	11 (100)	1.000
Mechanical ventilation	12 (12)	6 (54.5)	< 0.001
Non-invasive ventilation	88 (88)	5 (45.5)	
Pulmonary disease, yes	23 (23)	1 (9.1)	0.451
Asthma	15 (15)	1 (9.1)	
COPD	6 (6)		
Sarcoidosis	1 (1)		
**Smoker**			
No	75 (75)	9 (81.8)	0.797
Yes	4 (4)	0 (0)	
Previous	18 (18)	2 (18.2)	
Unknown	3 (3)		
Renal replacement therapy, yes	0 (0)	0 (0)	1.000
AKI, yes	63 (63)	0 (0)	< 0.001
Steroid treatment, yes	10 (10)	1 (9.1)	0.916
ACEi/ARB treatment, yes	37 (37)	2 (18.2)	0.332
Anticoagulant treatment, yes	22 (22)	5 (45.5)	0.132
Vasoactive treatment, yes	5 (5)	6 (54.5)	< 0.001
Antibiotic treatment, yes	60 (60)	8 (72.7)	0.527
SAPS-3	53 (47–58)	54 (49–58)	0.462
SOFA	6 (4–7)	6 (6–8)	0.885

Values are represented as median (IQR) or n (%). The p-value is calculated for continuous parameters with the Mann–Whitney U test, and for categorical parameters the chi-square test; p < 0.05 is considered significant.

*BMI* body mass index, *MAP* mean arterial pressure, *HIPEC* heated intraperitoneal chemotherapy, *COPD* chronic obstructive pulmonary disease, *ACEi/ARB* angiotensin-converting enzyme inhibitor/angiotensin receptor blockers, *SAPS* simplified acute physiology score, *SOFA* sequential organ failure assessment.

Physiological characteristics are also shown in Table 1. Between the COVID-19 and the non-COVID-19 patients, significant differences were observed in their clinical parameters, with respiratory rate, mean arterial pressure (MAP) and body temperature being higher in the COVID-19 group.

Malignancy represented the main indication for ICU admission for hyperthermic intraperitoneal chemotherapy (HIPEC) surgery in the non-COVID-19 group. On admission to the ICU, all patients in both groups required supplemental inspired oxygen, while the COVID-10 group and the non-COVID-19 group did not differ in their need for invasive ventilation during ICU stay (Supplementary Table S1). No significant differences were present in antibiotic use (Table 1). Supplementary Tables S2 and S3 present routine parameters measured for most COVID-19 patients at study inclusion. The partial pressure of arterial oxygen/fraction of inspired oxygen ratio (PaO2/FiO2), a clinical indicator of respiratory dysfunction, was strongly reduced and on average 143 mmHg (IQR 120–183 mmHg; normal range 400–500 mmHg) in the COVID-19 group. A significant rise in C-reactive protein (CRP), aspartate aminotransferase (AST), procalcitonin, lactate dehydrogenase (LDH), IL-6 and ferritin was seen in the COVID-19 patients, indicative of the ongoing inflammatory response and tissue damage. All these measures had the lower interquartile range above the reference range. D-dimer, indicative of fibrinolytic activation, was also above the reference range, while in the 20 patients where activated partial thromboplastin time (aPTT) was measured, the median aPTT value was within reference range.

### Detection of cfDNA, NE, Histone H3, GAS6 and sAXL in plasma

**Figure 1:**
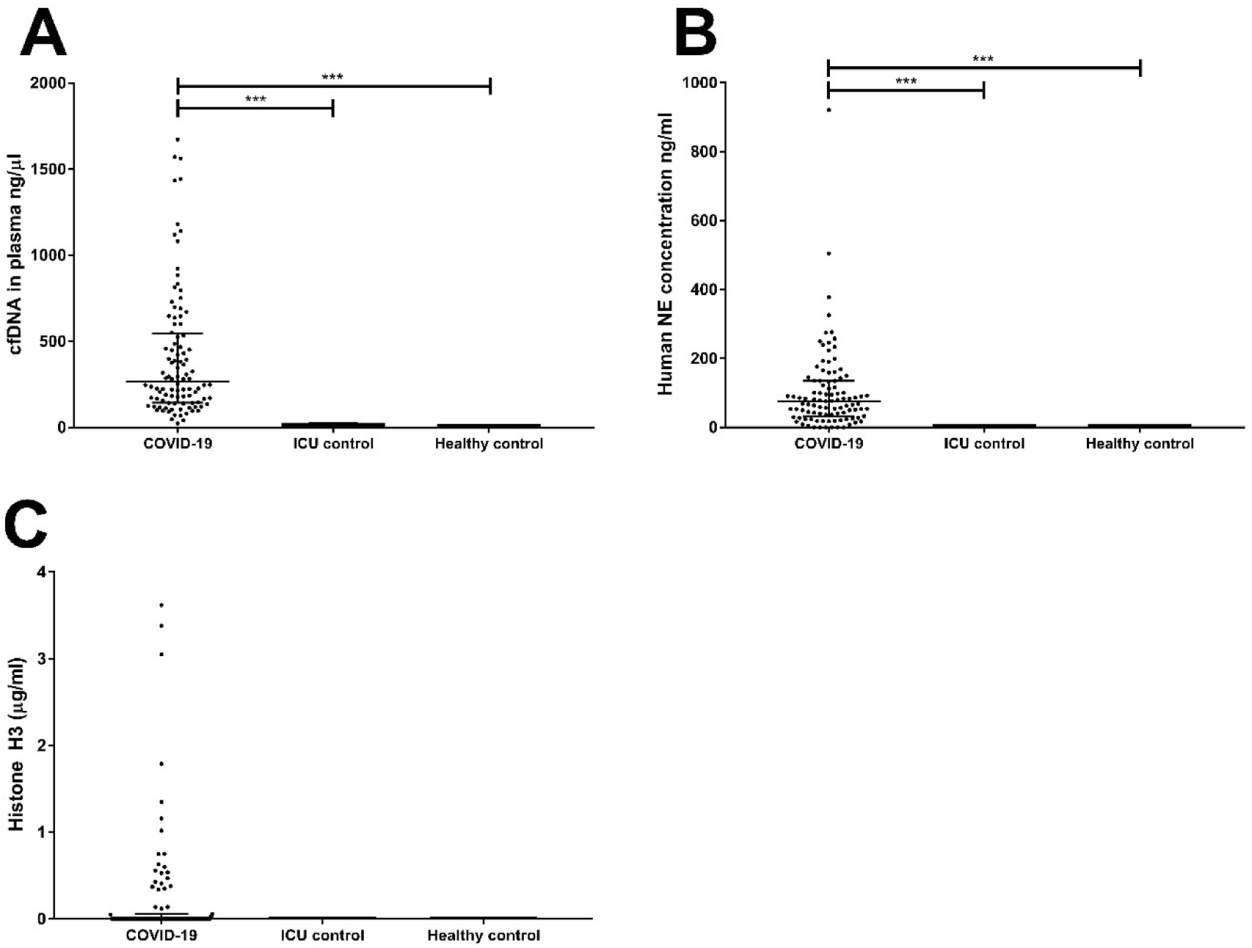
Detection of plasma markers cfDNA, NE, and Histone H3. Plasma from COVID-19 ICU patients (n = 100), non-COVID-19 ICU patients (n = 11), and healthy control (n = 15) was tested for the presence of cfDNA (**A**), neutrophil elastase (NE) (**B**), and extracellular histone H3 (**C**) at ICU admission. P-values were calculated with the Kruskal–Wallis test with Dunn’s post-hoc test. P-values were considered significant if p < 0.05; * 0.05, ** 0.01, *** 0.001. Statistical analysis of the three groups were p < 0.001 for cfDNA (**A**); p < 0.001 for NE (**B**) and p = 0.046 for histone H3 (**C**).

Plasma samples from the 111 ICU patients, were analyzed together with those of 15 healthy volunteers (Fig. 1). The cfDNA values from both ICU groups differ greatly (p < 0.001), with the COVID-19 group presenting 34-times higher levels than the control group (Fig. 1A). The levels of cfDNA did not differ between the ICU control and healthy control patients. There was a highly significant difference between both groups when NE was determined (p < 0.001; Fig. 1B) with NE being virtually absent from the ICU control group and the healthy controls. In the majority of COVID-19 patients (72%), no extracellular H3 could be detected at ICU admission. Further, no extracellular H3 was detected in samples from any of the ICU control patients, or healthy controls (Fig. 1C). Statistical analysis of the three groups by Kruskal-Wallace with Dunn’s post-hoc test showed a significant difference between the three groups (p = 0.046).

**Figure 2:**
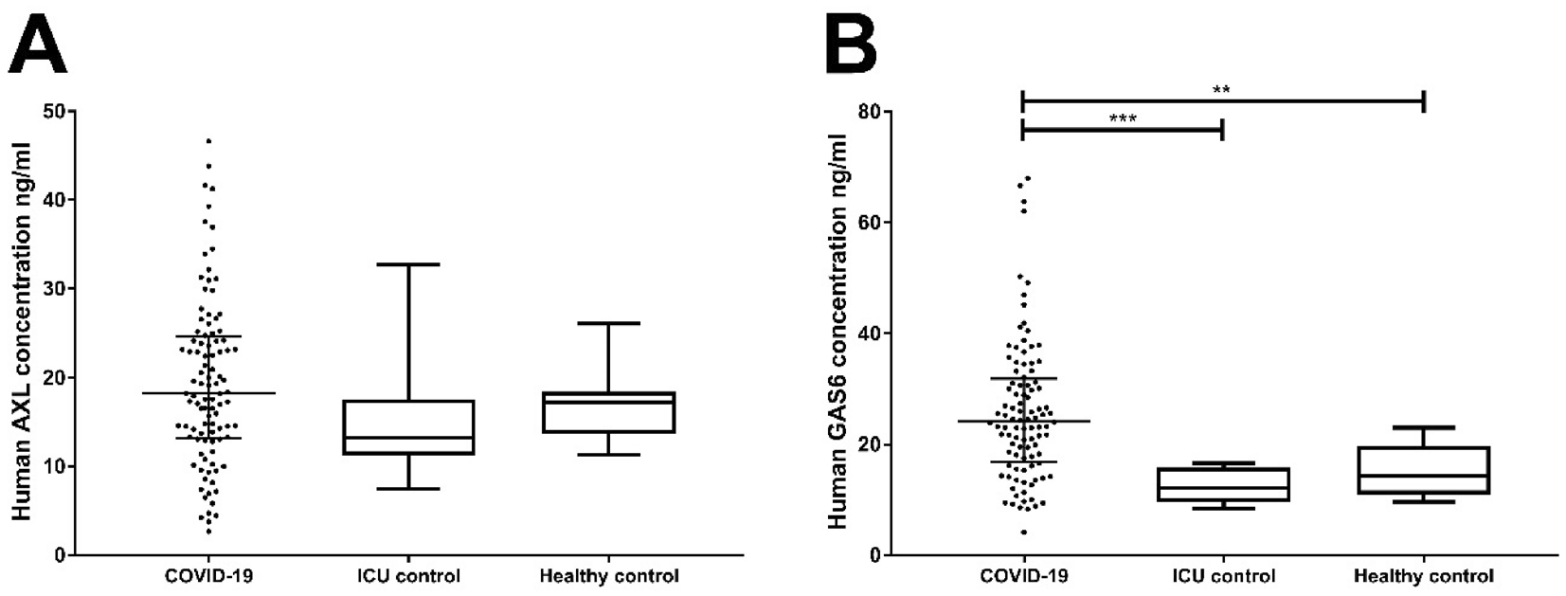
Detection of plasma markers GAS6 and sAXL. Plasma from COVID-19 IC patients (n = 100), non-COVID-19 IC patients (n = 11), and healthy controls (n = 15) was tested for the presence of GAS6 (**A**), and sAXL (**B**) at ICU admission. P-values were calculated with the Kruskal–Wallis test with Dunn’s post-hoc test. P-value were considered significant if p < 0.05; * 0.05, ** 0.01, *** 0.001. Statistical analysis of the three groups were p < 0.001 for Gas6 (**A**), and p = 0.134 for sAXL (**B**).

In COVID-19 and non-COVID-19 ICU patients, the GAS6 concentration at study inclusion was 24.2 (16.8–31.9) ng/mL and 12.1 (9.5–15.9) ng/mL respectively, indicating a significant difference between both groups (p < 0.001; Fig. 2A). The concentration of the healthy control group 14.4 (11.0–19.7) ng/mL is significantly lower (p < 0.001) compared to the COVID-19 ICU group, while no difference was found with the non-COVID-19 ICU group.

The concentration of sAXL at inclusion was higher for the COVID-19 group as compared to the ICU control group and the healthy controls (Fig. 2B), with levels being 18.2 (13.1–24.6) ng/mL, 13.2 (11.3–17.6) ng/mL and 17.3 (13.7–18.5) ng/mL respectively. None of the differences between these groups at time of inclusion were statistically significant.

### Correlations between different plasma markers and organ dysfunction at ICU admission

At ICU admission, cfDNA measured in the plasma of COVID-19 patients correlated with lactate dehydrogenase (LDH, 0.487; p < 0.001), which could indicate a common origin in cellular damage. The concentration of H3 correlated well (0.315; p < 0.001) with NE, and further correlations were observed with cfDNA. sAXL and NE concentrations (Table 2). sAXL correlated with GAS6 and procalcitonin. Remarkably, sAXL correlated significantly with the myocardial injury biomarker troponin I in this cohort.

**Table 2:** Correlations between various parameters measured at day 1.

	N	Correlation	*P*
cfDNA vs. Leukocytes	98	0.240	0.018
cfDNA vs. LDH	64	0.487	< 0.001
Histone H3 vs. cfDNA	100	0.223	0.026
Histone H3 vs. sAXL	100	0.225	0.025
Histone H3 vs. NE	99	0.315	0.001
sAXL vs Gas6	100	0.228	0.023
sAXL vs Troponin I	69	0.306	0.010
GAS6 vs procalcitonin	90	0.259	0.014
GAS6 vs heparin activitiy	11	- 0.726	0.011
NE vs fibrinogen	11	0.779	0.005

Correlations were calculated with the Spearman’s rank-order correlation test, only significant correlations are mentioned here.

*LDH* Lactate dehydrogenase.

Furthermore, we analysed whether the plasma values measured at ICU entry correlated with organ dysfunction that developed at a later stage of ICU stay. Table 3 illustrates that sAXL levels at day 1 were significantly lower in the patient group that developed a need for dialysis (p = 0.021). NE levels at ICU entry were significantly lower in patients who developed a thromboembolic event in the ICU. Remarkably, GAS6 concentrations at day 1 correlated with development of delirium, a manifestation associated with worse prognosis.

**Table 3:** Association between events during stay at the ICU and markers measured in COVID-19 patients.

Event	Analysis	Unit	Event present	Event not present	AUC	*P*
Dialysis			Yes (13)	No (87)		
*sAXL*	Day 1	ng/ml	11.7 (9.1–19.6)	19.3 (13.9–25.2)		0.021
	ROC curve				0.301 (0.158–0.443	0.029
VTE			Yes (14)	No (86)		
*NE*	Day 1	ng/ml	40.9 (20.0–66.2)	83.4 (40.7–143.9)		0.019
Delirium			Yes (9)	No (70)		
*Gas6*	Day 1	ng/ml	31.1 (23.8–38.5)	23.2 (16.2–30.3)		0.033
ICUAW			Yes (9)	No (91)		
*cfDNA*	Day 1	ng/µl	699.5 (273.1–1130.4)	246.6 (139.1–466.9)		0.019
	ROC curve				0.786 (0.566–0.909)	0.009

Indicated is the event and significant altered plasma markers. The difference at day 1 or the delta is calculated with Mann–Whitney U and the predictive power of an event by a marker is calculated by the AUC of the ROC curve. Values are considered significant if p < 0.05.

*VTE* various thromboembolic events, *ICUAW* Intensive Care Unit Acquired Weakness, *ROC* receiver operating characteristic, *AUC* area under the curve.

Lastly, cfDNA values correlated with ICUAW (intensive care unit acquired weakness) (p = 0.019). cfDNA concentrations were associated with development of ICUAW (AUC = 0.786; p = 0.009).

### Correlation of cfDNA levels and pulmonary function during ICU stay

**Figure 3:**
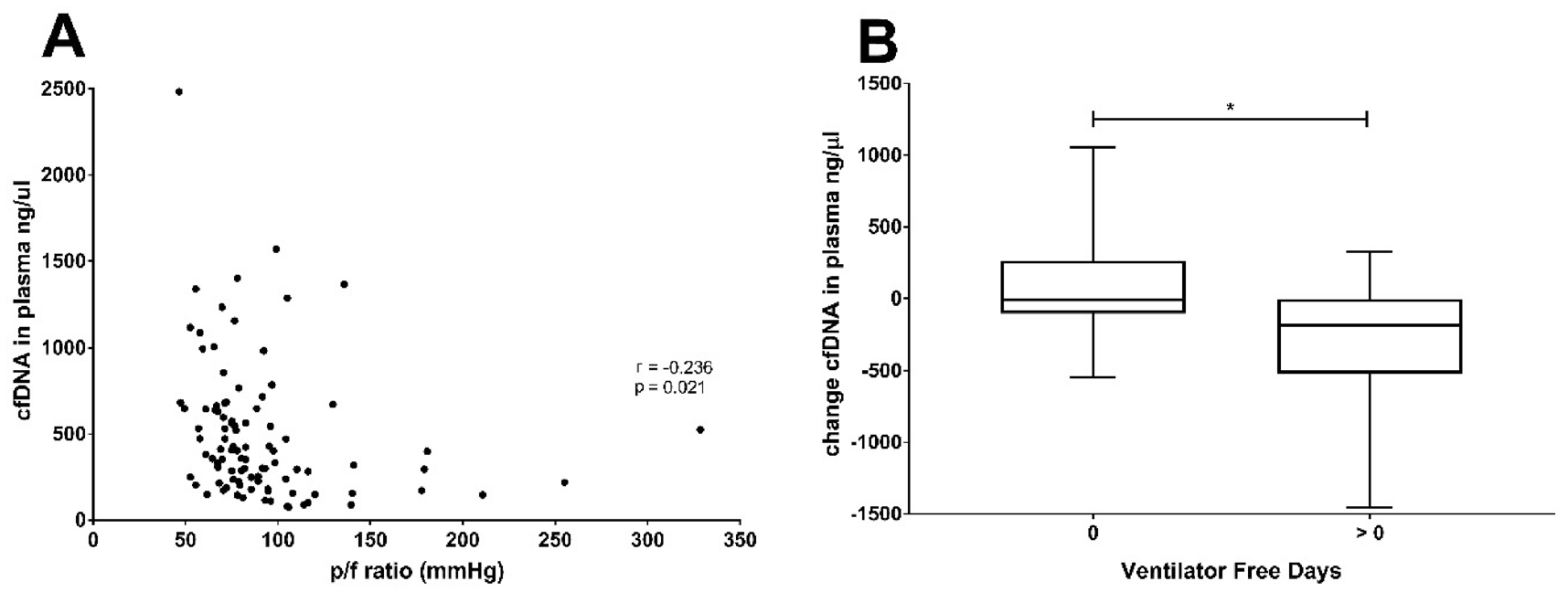
Correlations between pulmonary function and cfDNA in plasma. (**A**) The lowest p/f ratio measured during stay on the ICU and the cfDNA at ICU admission correlated significantly (r = -0.236; p = 0.021). Correlations were calculated with the Spearman’s rank-order correlation test. Correlations were considered significant if P < 0.05. (**B**) Change in level of cfDNA in COVID-19 patients on ICU and ventilator free days (VFD)**.** The change in the level of cfDNA in the plasma of COVID-19 patients was calculated by subtracting the average cfDNA level during late (> day 6) from the average level during early days (day 1–5). The patients were divided by VFD = 0 or > 0. P-values were calculated between the VFD groups divided by survival with the Mann–Whitney U test. P-value were considered significant if p < 0.05;*.

The linkage between cfDNA levels and clinical status appeared to reflect pulmonary function in the ICU patients that received invasive ventilation since we found that a significant correlation was found between the lowest pf-ratio during day 1 (Fig. 3A) and cfDNA levels during these periods (r = − 0.236; p = 0.021). The correlation was even stronger when the mean value of cfDNA in the early phase (days 1–5) was considered (r = − 0.393; p < 0.001). In line with this observation, the evolution of cfDNA levels was significantly linked to the ventilator-free days in COVID-19 patients during their stay at the ICU. Patients that required ventilation continuously, displayed a smaller decrease in cfDNA, compared to those that discontinued ventilation (Fig. 3B). The evolution of cfDNA from the early to the late phase correlated with the amount of ventilator-free days (r = − 0.356; p = 0.042). A similar correlation was found for the change in GAS6 concentration and ventilator-free days (r = − 0.377; p = 0.031).

### Prognosis and time-dependent parameter development in COVID-19

To study possible prognostic values of the baseline parameters determined here, we correlated these with patient outcome. The SAPS-3 (simplified acute physiology) score at ICU admission, but not the SOFA at this point in time, was significantly higher in the group of COVID-19 patients that did not survive (n = 24) compared to those that survived (n = 76; Supplementary Table S4). Non-survivors were older, hypertensive (79%), and had pre-existing vessel disease, malign disease or ischemic heart failure more frequently than survivors. Accordingly, they were more frequently treated with anticoagulants and angiotensin converting enzyme inhibitors/angiotensin-receptor blockers (ACEi/ARB) treatment. Acute kidney injury is more frequently found in non-survivors (p = 0.08). The PaO2/FiO2 ratio or need for mechanical ventilation was not different between both groups.

Although cfDNA, H3, NE, sAXL and GAS6 were all elevated in COVID-19 positive patients at admission, and some of these associated with development of organ dysfunction (see Table 3), linear regression analysis showed that none of these day-1 parameters was a good predictor of final outcome. Indeed, at ICU admission, there was no correlation of any of these parameters with SAPS-3 or SOFA scores.

To test whether change in parameters was informative, we studied the time-dependent development of the markers in 33 patients of whom we could obtain several samples, at admission and during ICU stay. We arbitrarily divided the results in an early (day 1–5) and a late (day 6–12) phase, using the mean value of the different samples obtained during each period (Supplementary Table S5). We inspected if the correlations observed in the initial samples persisted over the early (day 1–5) and late phase of ICU stay for the 33 COVID-19 patients for which data were available over this complete period of time (Supplementary Table S6). We observed that H3 correlated with NE over both time periods and generally showed persistent correlation with neutrophil counts. Strong correlations were seen between histone H3 levels and sAXL and neutrophils (0.673 and 0.631 respectively for early and late phase).

**Figure 4:**
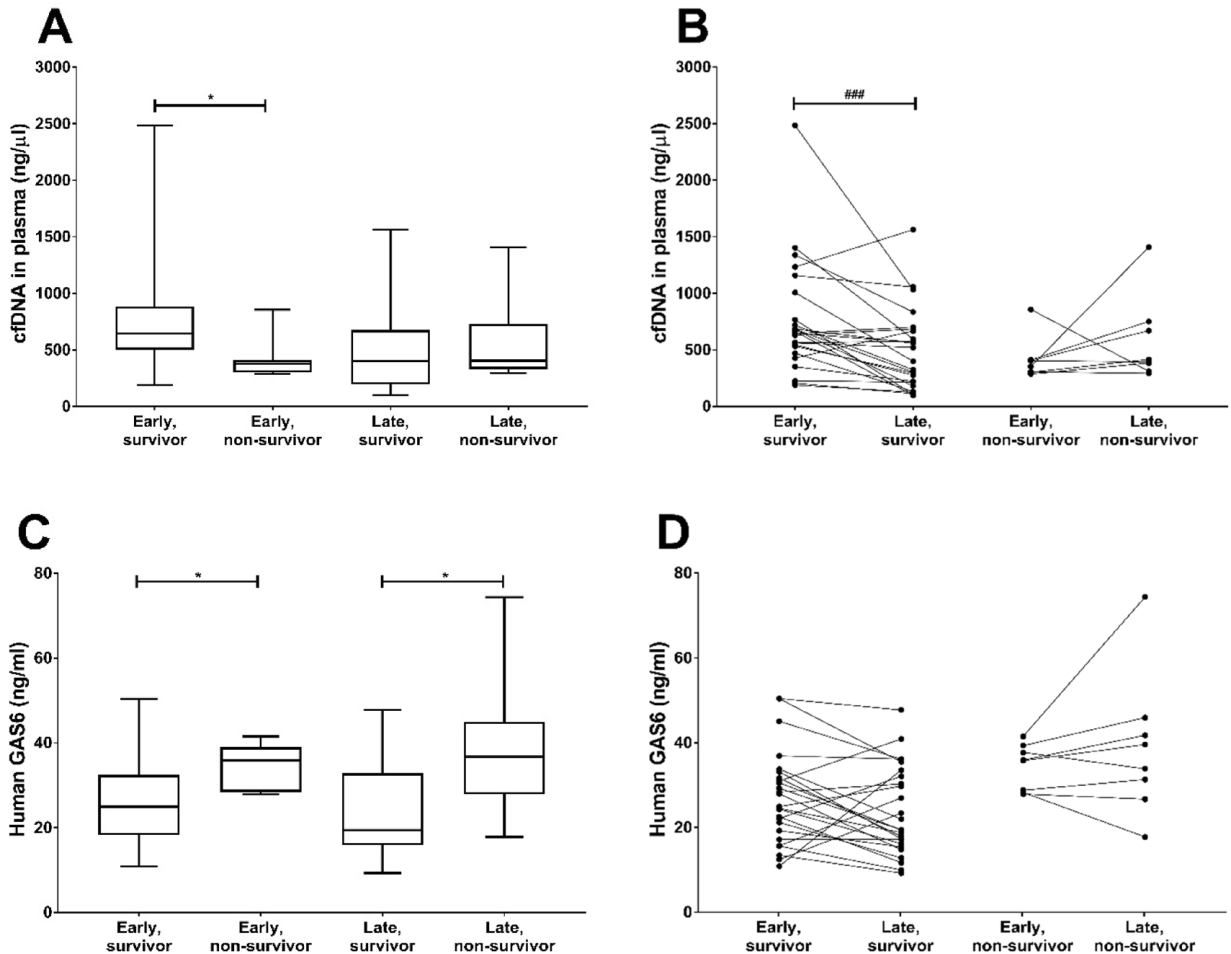
Sequential determination of plasma markers cfDNA and Gas6 in COVID-19 patients on ICU and 30-day mortality. Plasma from COVID-19 ICU patients was tested for the presence of cfDNA (**A**,**B**), and Gas6 (**C**,**D**) during early days (day 1–5), and during late days (> day 6). Survival is based on 30-day mortality. The average plasma marker levels were calculated per group for 33 patients (24 survivors and 8 non-survivors). P-values were calculated between survivors and non-survivors in both early and late days with the Mann–Whitney U test. P-value were considered significant if p < 0.05; * 0.05, ** 0.01, *** 0.001. P-values were calculated between early and late groups divided by survival with the Wilcoxon matched-pairs signed rank test. P-value were considered significant if p < 0.05; ^#^ 0.05, ^##^ 0.01, ^###^ 0.001.

Next, we divided the 33 patients according to their final outcome at 30 days (Fig. 4A). For those individuals who survived (n = 24), cfDNA levels were higher in the early samples as compared to the early samples from non-survivors (n = 8). However, in survivors there was a significant 38% decrease in cfDNA concentration in the late plasma samples (Fig. 4A,B). Further, in the surviving patients, the GAS6 concentration was significantly lower than in the non-survivors both in early and late samples, and decreased more than 22% in late samples in the survivors (Fig. 4C,D). In contrast, neither cfDNA nor GAS6 decreased in the group of non-survivors (n = 8), with their late values being very similar or higher to the early ones. Similar trends were observed in H3 and NE measurements, although without reaching significance.

**Figure 5:**
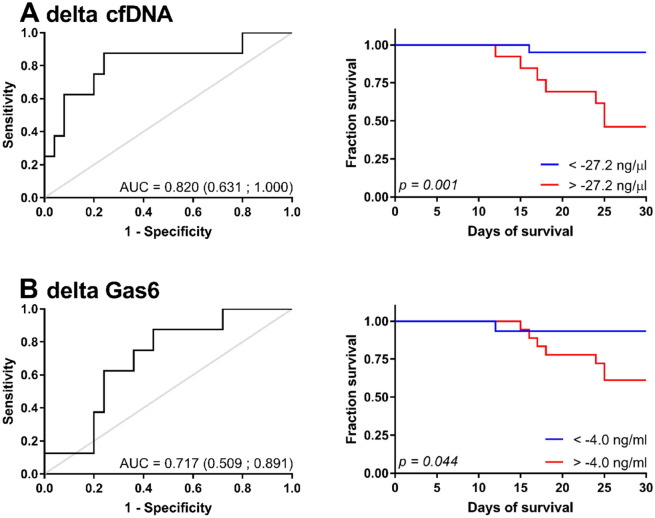
Ideal cutoff value and Kaplan–Meier curves for the prediction of 30-day survival based on sequential levels of (**A**) cfDNA and (**B**) Gas6. Receiver operating characteristic (ROC) curve analysis identified the ideal cut-off value with the Youden index of the difference in cfDNA and Gas6 levels in plasma between the early and late time group (n = 33). Kaplan–Meier survival curves for 30-day survival were created based on the identified cutoff value. P-value was calculated with the Mantel-Cox test.

When we analysed the data by means of ROC and Kaplan–Meier analysis we found that the change in cfDNA concentration from early to late samples is able to significantly predict mortality, with a cut-off value of − 27.22 ng/mL (AUC: 0.820 (0.631–1.000), p = 0.001; Fig. 5A). This cut-off value resulted in a sensitivity of 87.5%, specificity of 76%, positive predictive value (PPV) of 54% and negative predictive value (NPV) of 95%. Similarly, GAS6 change predicted mortality when a cut off value of − 4.03 ng/mL was used (AUC: 0.717 (0.509–0.891), p = 0.044, Fig. 5B). This cut-off value resulted in a sensitivity of 87.5%, specificity of 56%, PPV of 39% and NPV of 93%.

## Discussion

Our study broadly assessed a series of biomarkers reflecting the process of cellular damage and NETosis as well as a mechanism of early response to damage, the GAS6/AXL pathway, in a group of severely ill patients, consecutively admitted at an ICU during the COVID-19 pandemic. A non-COVID-19 group was included consisting of ICU patients suffering from malignancies requiring surgery. Respiratory illness and acute respiratory distress syndrome (ARDS) were the major effects caused by COVID-19 with PaO2/FiO2 ratios being clearly lower in the COVID-19 group, resulting in higher percentage of invasive respiration applied. Increased tissue damage, and particularly lung tissue damage, with more pronounced inflammation, in combination with pulmonary thromboembolic disease could explain this difference. This is underscored by increases in CRP, IL-6, D-dimer, LDH, ferritin, procalcitonin, ASAT and increased neutrophil counts at admission (Supplementary Tables S2 and S3).

Already at admission to the ICU, levels of cfDNA, H3 and NE differed significantly between the COVID-19 and non-COVID-19 groups. This is likely indicative of increased tissue damage and neutrophil activation in this group. None of these parameters measured at day 1 predicted outcome, however some did correlate with organ dysfunction (see Table 3), an observation which became more evident when we analyzed samples obtained on different days during the course of the ICU stay, available from a subset of patients (n = 33). We noted that change in cfDNA and GAS6 levels correlated with outcome, indicating that the development over time in these parameters holds more information on mortality than the levels at day 1 alone. Day 1 samples provide a snapshot of ongoing pathomechanisms, but changes in parameters are possibly better able to catch the underlying dynamics and indicate a course towards recovery or a worsening disease state.

The importance of studying parameter dynamics is illustrated by the observation that changes in cfDNA and GAS6 significantly correlated with function measured as ventilator-free days at ICU. Moreover, the change in these parameters predicted mortality. Changes in these two parameters, cfDNA and GAS6 are indicative of an underlying interplay between the immune system and organ damage, contributing to mortality in COVID-19.

It has been suggested that cfDNA could serve as a surrogate marker for NETosis in critically ill mechanically ventilated patients in whom NETs contribute to local alveolar inflammation^53^. Indeed, a crucial role of NETosis in the infection and pathological course has been proposed in COVID-19, inducing immunothrombosis and complement activation^17,18,54^. However, our data show that while the correlation of NE and histone H3 was strong and consistent in time (Table 2 and Supplementary Table S6); their correlation with cfDNA was less significant. This could indicate that in COVID-19 NET formation is better reflected by the release of neutrophil-specific markers such as NE or MPO/DNA complexes, while cfDNA could relate to cellular damage in a broader sense. cfDNA is a DAMP able to activate TLRs. The sustained increase in cfDNA observed in COVID-19 patients would propagate inflammation through Toll like receptor (TLR) activation. Increased levels of NE are able to reduce the lung permeability barrier function and induce release of pro-inflammatory cytokines, collectively inducing emphysematous lesions^55^. However, in our hands, the measurement of cfDNA correlated better with parameters of the disease and its evolution was predictive of disease outcome.

We observed H3 positive samples levels at ICU admission, like we found earlier in a critically ill ICU population of sepsis patients^50^. While this points at similar pathways being involved in disease onset and progression, other reports^56^ pointed out that COVID-19 clinical features are similar but different from those seen in sepsis. The observed normal platelet counts found in most samples in this study, irrespective of the phase of the disease or its outcome, further underscore this point. A possible early onset of NETosis, with an associated rise in extracellular histones is supported by our observation that NE levels were evident already from day 1 till day 12, implying neutrophil activation and NETosis, accompanied by cfDNA also being present already at day 1.

In our COVID-19 cohort, GAS6 concentrations at ICU admission more than doubled those of non-COVID-19 IC patients. Among plasma determinations that correlate with GAS6 are the interleukins IL-6^41,42^ and IL-8^42^. GAS6, IL-6 and IL-8 concentrations are increased in septic patients who develop acute lung injury (ALI;^41^. Plasma GAS6 concentration was able to significantly discriminate patients that would develop ALI in that cohort^43^. Also, non-survivors of sepsis in ICU tend to have initial higher concentration of GAS6 and GAS6 could predict mortality with an area under the curve (AUC) of 0.7^42^. However, in our cohort, no evident correlation with severity of the lung symptoms could be established, which could reflect a specific characteristic of SARS-CoV-2 interaction with the system.

In our study group, GAS6 concentration was maximal at IC admission, and remained high in non-survivors. Ni et al. have shown that recombinant GAS6 infusion improves the outcome of experimental sepsis in mice, controlling multi-organ dysfunction^57^. One of the target cells of GAS6 in bacterial infection is the vascular endothelium, which showed reduced LPS-induced permeability in the presence of GAS6. Interestingly, GAS6 is also necessary to maintain the response of vascular endothelium during inflammatory conditions, allowing endothelial cell interactions with platelets and leukocytes^39^.

While the concentration of sAXL was found increased in sepsis cohorts^41,42^, the increase was not so evident and did not significantly correlate with organ damage, similarly to our observation in COVID-19 patients. Soluble AXL is an disintegrin and metalloproteinase (ADAM)-shed form of the receptor, found in plasma in a complex with GAS6^58^. The specific increase of GAS6 over sAXL in COVID-19 could reflect a need of free, active GAS6 in this condition. Of note, we observed correlation between sAXL and plasma creatinine in both the early and late phase of ICU admission with correlations of 0.412 (p = 0.016) and of 0.465 (p = 0.007) respectively, hinting at a contribution of sAXL to renal function.

### Limitations

The present study characterizes samples of COVID-19 patients from the time they were admitted to the ICU, on average 10 days after onset of symptoms. Samples from earlier stages of the disease might show initial differences in the biomarkers studied. Furthermore, all patients studied here were severely ill, and therefore it was difficult to discriminate outcomes in a homogenously affected population. Finally, sequential samples were available in only a fraction of patients (33 out of 100). Still, this group was informative, showing that indication of prolonged NETs-formation was related to poor outcome and respiratory dysfunction, suggesting a detrimental effect of this process in severe COVID-19. The control ICU group is relatively small and uniformly admitted for HIPEC surgery, this limits the ability to extrapolate to other ICU populations. In addition, given the absence of infectious disease in the ICU control group and healthy individuals, care should be taken to not interpret our data as being characteristic for exclusively COVID-19 patients, as they may represent characteristics of a more general type of critically infected patient. Taking into account the smaller group size allows correct statistics, the availability of clinical and lab parameters of the non_COVID-19 population however does facilitate comparison to other ICU populations.

## Conclusion

We have shown the presence of cfDNA and NE in plasma of COVID-19 patients to be significantly different from plasma of ICU patients who did not have COVID-19 or healthy controls. Extracellular histone H3 was exclusively found in COVID-19 patients and not in non-infectious ICU controls or healthy controls. These increases could reflect, at least in part, the formation of NETs during disease progression. An increase of the GAS6 immunomodulatory vitamin K dependent protein was detected.

Although the markers tested at ICU admission did not predict outcome, the evolution of cfDNA and GAS6 differentiated survivors and non-survivors, with decreasing levels correlating with survival. The involvement of NETosis and DAMPS in COVID-19 provides a possible rational basis for treatment options that are able to target NETosis or its associated cytotoxic and pro-inflammatory DAMPS in support of existing therapies in the ICU.

## Supplementary Information

Supplementary Tables.

## Data Availability

The data used and/or analyzed in the present study are available from the corresponding author on reasonable request.
