# Supplementary material for: Evolution of NETosis markers and DAMPs have prognostic value in critically ill COVID-19 patients
Source: Sci Rep. 2021 Aug 3;11:15701. doi: 10.1038/s41598-021-95209-x (PMC8333321; doi:10.1038/s41598-021-95209-x)
Supplement: Supplementary file 1 — Supplementary Tables. [file 41598_2021_95209_MOESM1_ESM.docx]

**Supplementary Tables**

**Evolution of NETosis markers and DAMPs have prognostic value in critically ill COVID-19 patients**

Joram Huckriede^1^, Sara Bülow Anderberg^2^, Albert Morales^3^, Femke de Vries^1^, Michael Hultström^2,4^, Anders Bergqvist^5^, José T. Ortiz^6^, Jan Willem Sels^7,8^, Kanin Wichapong^1^, Miklos Lipcsey^2,9^, Marcel van de Poll^7,10^, Anders Larsson^11^, Tomas Luther^2^, Chris Reutelingsperger^1^, Pablo Garcia de Frutos^12^, Robert Frithiof^#2^, Gerry A.F. Nicolaes^1#*^

^1^Department of Biochemistry, Cardiovascular Research Institute Maastricht (CARIM), Maastricht University, Maastricht, the Netherlands

^2^Department of Surgical Sciences, Section for Anaesthesia & Intensive Care

Uppsala University, Uppsala, Sweden

^3^Department of Cell Death and Proliferation, IIBB-CSIC, IDIBAPS, and BCLC, CIBEREHD, Barcelona, Spain

^4^Department of Medical Cell Biology, Integrative Physiology, Uppsala University, Uppsala, Sweden

^5^Department of Medical Sciences, Clinical Microbiology, Uppsala University, Uppsala, Sweden.

^6^Cardiology Department, Hospital Clinic Barcelona and CIBERCV, Spain

^7^Department of Intensive Care Medicine, Maastricht University Medical Centre MUMC+), Maastricht, the Netherlands

^8^Department of Cardiology, Maastricht University Medical Centre (MUMC+), Maastricht, the Netherlands

^9^Hedenstierna laboratory, Anaesthesiology and Intensive Care Medicine, Department of Surgical Sciences, Uppsala University, Uppsala, Sweden.

^10^Department of Surgery, Maastricht University Medical Centre (MUMC+), School for Nutrition and Translational Research in Metabolism (NUTRIM), Maastricht University

^11^Department of Medical Sciences, Clinical Chemistry, Uppsala University, Uppsala, Sweden.

^12^Department of Cell Death and Proliferation, IIBB-CSIC, IDIBAPS and CIBERCV, Barcelona, Spain;

^#^ These authors contributed equally

^*^corresponding author:

Dr. G.A.F. Nicolaes, Department of Biochemistry, Cardiovascular Research Institute Maastricht (CARIM), Maastricht University. P.O. Box 616, 6200 MD Maastricht, the Netherlands. Tel. +31-43-3881688; Fax. +31-43-3884159

e-mail: g.nicolaes@maastrichtuniversity.nl

**Supplementary Table 1: Registered clinical events of patients during stay on the ICU.**

|  | **ICU COVID-19 n = 100** | **ICU Control n = 11** | ***P*** |
| --- | --- | --- | --- |
| **Invasive Ventilation,** yes | 59 (59) | 6 (54.5) | 0.760 |
| **Thromboembolic event,** yes | 14 (14) | 0 (0) | < 0.001 |
| Stroke | 4 |  |  |
| Myocardial infarction | 1 |  |  |
| Pulmonary embolism | 9 |  |  |
| **Bleeding event,** yes | 3 (3) |  |  |
| **ICUAW,** yes | 9 (9) |  |  |
| **Delirium,** yes | 9 (9) |  |  |
| **Secondary infection**, yes | 55 (55) | 0 (0) | < 0.001 |
| **Worst p/f ratio** | 78.8  (67.7 – 97.3) | 271.5  (227.3 – 373.9) | <0.001 |
| **p/f ratio < 150 mmHg** | 94 | 0 | <0.001 |
| **ARDS** 0 | 3 | 2 (18.2) | <0.001 |
| 1 | 8 | 2 (18.2) |  |
| 2 | 40 | 1 (9.1) |  |
| 3 | 40 |  |  |
| Unknown | 9 | 6 (54.5) |  |
| **AKI,** yes | 60 | 0 | <0.001 |
| **Severe AKI (≥3),** yes | 17 |  |  |
| **Renal replacement therapy,** yes | 13 (13) |  |  |
| **Vasoactive treatment,** yes | 60 (60) |  |  |
| **Chloroquine,** yes | 11 |  |  |
| **Hydroxychloroquine,** yes | 2 |  |  |
| **Tocilizumab,** yes | 2 |  |  |
| **Remdesivir,** yes | 0 |  |  |
| **Betamethasone,** yes | 2 |  |  |
| **Length ICU stay,** days | 10 (6 – 17) |  |  |
| **Vasoactive free days** | 25 (11 – 30) |  |  |
| **Ventilator free days** | 22 (0 – 30) |  |  |
| **Dialysis free days** | 30 (10 – 30) |  |  |
| **Alive at discharge** | 80 | 11 | 0.091 |
| **Alive at 30-days** | 76 |  |  |
| **Alive at 90-days** | 71 |  |  |

Data is presented as n (%). The p-value is calculated for continuous parameters with the Mann-Whitney U test, and for categorical parameters the chi-square test; p < 0.05 is considered significant.

ICUAW (Intensive Care Unit Acquired Weakness)

**Supplementary Table 2: Baseline laboratory profile COVID-19 and non-COVID-19 ICU patients expressed as median (IQR).**

|  |  | **Normal range** | **n** | **ICU COVID-19** | **n** | **ICU Control** | ***P*** |
| --- | --- | --- | --- | --- | --- | --- | --- |
| **pO2/FiO2** | mmHg | 400-500 | 93 | 143  (120 – 183) | 5 | 271  (227 – 374) | < 0.001 |
| **Leukocytes** | 10^9^/L | 3.5 – 9.0 | 98 | 7.6  (6.2 – 10.1) | 4 | 12.5  (9.7 – 15.7) | 0.020 |
| **Erythrocytes** | 10^12^/L | 4.30 – 5.70 | 79 | 4.16  (3.73 – 4.63) | 4 | 3.73  (2.94 – 4.33) | 0.207 |
| **Platelets** | 10^9^/L | 150 – 350 | 97 | 211  (156 – 300) | 4 | 358  (233 – 457) | 0.054 |
| **CRP** | mg/L | < 5.0 | 97 | 169  (122 – 230) | 4 | 4.9  (3.1 – 44.2) | < 0.001 |

P-value is calculated with the Mann-Whitney U test, p-values < 0.05 are considered significant.

CRP (C-reactive Protein)

**Supplementary Table 3: Baseline laboratory profile of COVID-19 ICU patients expressed as median (IQR).**

|  |  | **Normal range** | **n** | **ICU COVID-19** |
| --- | --- | --- | --- | --- |
| **Neutrophils** | 10^9/L | 1.3 – 5.4 | 70 | 5.8 (3.8 – 7.5) |
| **Lymphocytes** | 10^9/L | 0.7 – 3.9 | 76 | 1.0 (0.8 – 1.2) |
| **Monocytes** | 10^9/L | 0.1 – 0.8 | 66 | 0.3 (0.2 – 0.5) |
| **Basophiles** | 10^9/L | 0.0 – 0.1 | 20 | 0.0 (0.0 – 0.04) |
| **Eosinophiles** | 10^9/L | 0.0 – 0.5 | 51 | 0.0 (0.0 – 0.03) |
| **MCV** | fL | 82.0 – 98.0 | 97 | 88.2 (84.0 – 92.5) |
| **ALT** | µkat/L | 0.15 – 1.10 | 73 | 0.65 (0.46 – 1.09) |
| **AST** | µkat/L | 0.25 – 0.75 | 71 | 1.09 (0.76 – 1.76) |
| **Procalcitonin** | µg/L | < 0.05 | 90 | 0.49 (0.17 – 1.10) |
| **LDH** | µkat/L | 1.8 – 3.4 | 64 | 5.6 (4.8 – 6.9) |
| **IL-6** | ng/L | < 7.0 | 61 | 103 (53 – 167) |
| **Ferritin** | µg/L | 25 - 310 | 67 | 1168 (517 – 2571) |
| **Fibrin, D-Dimer** | mg/L | < 0.66 | 92 | 1.40 (0.90 – 2.59) |
| **Troponin I** | ng/L | < 35.0 | 69 | 24.0 (9.9 – 54) |
| **APTT** | sec | 30 – 42 | 20 | 42 (36 – 50) |
| **Creatinine** | µmol/L | 60 - 105 | 96 | 81 (62 – 97) |

P-value is calculated with the Mann-Whitney U test, p-values < 0.05 are considered significant.

MCV (Mean Cell Volume), ALT (Alanine Aminotransferase), AST (Aspartate Transaminase), LDH (Lactate Dehydrogenase), IL-6 (Interleukin-6), APTT (Activated Partial Thromboplastin Time)

**Supplementary Table 4: Survivors vs non-survivors comparison of the baseline.**

|  | **ICU Covid-19 Survivor  n = 76** | **ICU Covid-19 Non Survivor n = 24** | ***P*** |
| --- | --- | --- | --- |
| **Age,** yrs, median | 57 (50 – 69) | 72 (67 – 76) | < 0.001 |
| **Gender,** male N | 55 (72.4) | 19 (79.2) | 0.508 |
| **BMI** | 29.0 (26.2 – 33.7) | 27.0 (23.9 – 31.1) | 0.098 |
| **Respiratory rate,** breaths/min | 28 (22 – 35) | 28 (23 – 38) | 0.682 |
| **Heart rate,** beats/min | 90 (78 – 100) | 84 (73 – 103) | 0.188 |
| **MAP,** mmHg | 90 (81 – 97) | 85 (75 – 94 | 0.246 |
| **Temperature,** °C | 38.0 (37.4 – 38.7) | 38.1 (37.5 – 38.8) | 0.834 |
| **Diabetes,** yes | 21 (27.6) | 8 (33.3) | 0.592 |
| **Hypertension,** yes | 33 (43.4) | 19 (79.2) | 0.002 |
| **Heart failure,** yes | 3 ( 3.9) | 2 (8.3) | 0.591 |
| **Ischemic heart failure,** yes | 6 (7.9) | 6 (25.0) | 0.035 |
| **Vessel disease,** yes | 8 (10.5) | 9 (37.5) | 0.004 |
| **Malign disease,** yes | 2 (2.6) | 4 (16.7) | 0.028 |
| **HIPEC surgery,** yes | 0 (0) | 0 (0) | 1.000 |
| **PaO2/FiO2-ratio**, mmHg | 138 (118 – 178) | 126 (110 – 179) | 0.330 |
| **Ventilation,** yes | 76 | 24 |  |
| Mechanical ventilation | 8 (10.5) | 4 (16.7) | 0.420 |
| Non-invasive ventilation | 68 (89.5) | 20 (83.3) |  |
| **Pulmonary disease,** yes | 16 (21.1) | 7 (29.2) | 0.410 |
| Asthma | 12 (15.8) | 3 (12.5) |  |
| COPD | 3 (3.9) | 3 (12.5) |  |
| Sarcoidosis |  | 1 (4.2) |  |
| **AKI,** yes | 44 (57.9) | 19 (79.2) | 0.080 |
| **Smoker**  No | 61 (80.3) | 14 (58.3) | 0.261 |
| Yes | 2 (2.6) | 2 (8.3) |  |
| Previous | 13 (17.1) | 5 (20.8) |  |
| Unknown | 0 | 3 (12.5) |  |
| **Renal replacement therapy,** yes | 0 (0) | 0 (0) | 1.000 |
| **AKI**, yes | 41 (53.9) | 19 (79.2) | 0.033 |
| **Steroid treatment,** yes | 8 (10.5) | 2 (8.3) | 0.741 |
| **ACEi/ARB treatment,** yes | 21 (27.6) | 16 (66.7) | 0.001 |
| **Anticoagulant treatment,** yes | 11 (14.5) | 11 (45.8) | 0.001 |
| **Vasoactive treatment,** yes | 2 (2.6) | 3 (12.5) | 0.090 |
| **Antibiotic treatment**, yes | 46 (60.5) | 14 (58.3) | 0.793 |
| **SAPS-3** | 51 (46 – 56) | 61 (53 – 69) | < 0.001 |
| **SOFA** | 5 (4 – 7) | 7 (5 – 9) | 0.214 |

Baseline characteristics of COVID-19 ICU patients at admission to ICU, divided by 30-day survival. Data is represented as median (IQR) or n (%). The p-value is calculated for continuous parameters with the Mann-Whitney U test, and for categorical parameters
the chi-square test; p < 0.05 is considered significant.
BMI (Body Mass Index), MAP (Mean Arterial Pressure), HIPEC (Heated Intraperitoneal Chemotherapy), COPD (Chronic Obstructive Pulmonary Disease), AKI (Acute Kidney Injury), ACEi/ARB (Angiotensin-Converting Enzyme inhibitor/ Angiotensin Receptor Blockers), SAPS (Simplified Acute Physiology Score), SOFA (Sequential Organ Failure Assessment)

**Supplementary Table 5: Comparison of averages between COVID-19 ICU patient parameters obtained during early (days 1-5) and late (days 6-12) stay at the ICU.**

| **Parameters** | **Normal range** | **Early** | **Late** | ***P*** |
| --- | --- | --- | --- | --- |
| cfDNA | < 6.0 | 563.7  (378.5 – 741.3) | 398.8  (250.0 – 677.3) | 0.018 |
| Histone H3 | < 0.01 | 0.08  (0.00 – 0.51) | 0.01  (0.00 – 0.35) | 0.716 |
| NE |  | 87.2  (56.5 – 126.5) | 100.4  (52.8 – 174.1) | 0.339 |
| GAS6 | 13 – 23 | 28.4  (21.7 – 35.9) | 26.7  (17.3 – 35.8) | 0.228 |
| sAXL | 24 – 72 | 18.2  (11.7 – 23.2) | 20.3  (12.7 – 25.9) | 0.044 |
| Leukocytes | 3.5 – 9.0 | 7.9  (6.4 – 10.1) | 11.7 (8.7 – 14.2) | < 0.001 |
| Erythrocytes | 4.30 – 5.70 | 4.18  (3.82 – 4.28) | 3.60 (3.38 – 4.01) | < 0.001 |
| Platelets | 150 – 350 | 248 (178 – 303) | 430 (317 – 534) | < 0.001 |
| MCV | 82.0 – 98.0 | 89.6  (85.7 – 92.8) | 91.6 (88.0 – 94.8) | < 0.001 |
| ALT | 0.15 – 1.10 | 0.71  (0.52 – 0.95) | 1.04 (0.76 – 1.52) | < 0.001 |
| AST | 0.25 – 0.75 | 1.12 (0.87 – 1.53) | 1.71 (1.17 – 2.27) | < 0.001 |
| Creatinine | 60 - 105 | 84.7  (64.9 – 110.1) | 108.7 (66.8 – 202.8) | 0.001 |
| Lymphocytes | 0.7 – 3.9 | 0.9 (0.6 – 1.2) | 1.0 (0.7 – 1.6) | 0.004 |
| Monocytes | 0.1 – 0.8 | 0.3 (0.3 – 0.5) | 0.6 (0.1 – 0.9) | 0.059 |
| Eosinophils | 0.0 – 0.5 | 0.1  (0.0 – 0.1) | 0.1  (0.0 – 0.2) | 0.050 |

Values are represented as median (IQR) and difference is calculated with the related samples Wilcoxon signed rank test. P-values < 0.05 are considered significant.

MCV (Mean Cell Volume), ALT (Alanine Aminotransferase), AST (Aspartate Transaminase)

**Supplementary Table 6: Correlations between measured plasma levels of
cfDNA, NE, sAXL, GAS6, and various parameters measured during the average of early (days 1-5) or late (days 6-12) in COVID-19 ICU patients, n = 33.**

|  | **N** | **Correlation** | ***P*** |
| --- | --- | --- | --- |
| **Early** |  |  |  |
| cfDNA vs Leukocytes | 33 | 0.398 | 0.022 |
| cfDNA vs Neutrophils | 27 | 0.428 | 0.026 |
| NE vs sAXL | 33 | 0.415 | 0.016 |
| Histone H3 vs NE | 33 | 0.438 | 0.011 |
| Histone H3 vs sAXL | 33 | 0.673 | < 0.001 |
| Histone H3 vs Ferritin | 22 | 0.588 | 0.004 |
| Histone H3 vs Bilirubin | 33 | 0.511 | 0.002 |
| sAXL vs GAS6 | 33 | 0.407 | 0.019 |
| sAXL vs Creatinine | 33 | 0.412 | 0.017 |
| sAXL vs SOFA | 33 | 0.415 | 0.016 |
| **Late** |  |  |  |
| cfDNA vs Eosinophils | 15 | 0.565 | 0.028 |
| cfDNA vs SOFA | 32 | 0.562 | 0.002 |
| Histone H3 vs NE | 33 | 0.437 | 0.011 |
| Histone H3 vs Eosinophils | 15 | 0.563 | 0.029 |
| Histone H3 vs Neutrophils | 16 | 0.631 | 0.009 |
| Histone H3 vs Creatinine | 32 | 0.409 | 0.020 |
| GAS6 vs Troponin I | 31 | 0.394 | 0.028 |
| GAS6 vs Bilirubin | 32 | 0.503 | 0.003 |
| sAXL vs Bilirubin | 32 | 0.402 | 0.007 |
| sAXL vs Creatinine | 32 | 0.465 | 0.007 |
| sAXL vs SOFA | 32 | 0.412 | 0.019 |

Correlations were calculated with the Spearman’s rank-order correlation test, only significant correlations are mentioned here.

SOFA (Sequential Organ Failure Assessment)

s
